# Population structure of *Wolbachia* and cytoplasmic introgression in a complex of mosquito species

**DOI:** 10.1186/1471-2148-13-181

**Published:** 2013-09-03

**Authors:** Emilie Dumas, Célestine M Atyame, Pascal Milesi, Dina M Fonseca, Elena V Shaikevich, Sandra Unal, Patrick Makoundou, Mylène Weill, Olivier Duron

**Affiliations:** 1 Institut des Sciences de l’Evolution, UMR5554 CNRS, Université Montpellier 2, 34095 Montpellier cedex 05, France; 2 Université de la Réunion-CRVOI (Centre de Recherche et de Veille sur les Maladies Emergentes dans l‘Océan Indien), 2 rue Maxime Rivière, 97490 Sainte Clotilde, France(Ile de La Réunion; 3 Center for Vector Biology, Rutgers University, 180 Jones Av, New Brunswick NJ 08901, USA; 4 N.I. Vavilov Institute of General Genetics, Russian Academy of Sciences, Gubkin Str. 3, Moscow 119991, Russia

**Keywords:** Endosymbiosis, *Wolbachia*, Cytoplasmic introgression, *Culex pipiens* complex

## Abstract

**Background:**

The maternally inherited bacterium *Wolbachia* often acts as a subtle parasite that manipulates insect reproduction, resulting potentially in reproductive isolation between host populations. Whilst distinct *Wolbachia* strains are documented in a group of evolutionarily closely related mosquitoes known as the *Culex pipiens* complex, their impact on mosquito population genetics remains unclear. To this aim, we developed a PCR-RFLP test that discriminates the five known *Wolbachia* groups found in this host complex. We further examined the *Wolbachia* genetic diversity, the variability in the coinherited host mitochondria and their partitioning among members of the *Cx. pipiens* complex, in order to assess the impact of *Wolbachia* on host population structure.

**Results:**

There was a strong association between *Wolbachia* and mitochondrial haplotypes indicating a stable co-transmission in mosquito populations. Despite evidence that members of the *Cx. pipiens* complex are genetically distinct on the basis of nuclear DNA, the association of *Wolbachia* and mtDNA with members of the *Cx. pipiens* complex were limited. The *Wolbachia w*Pip-I group, by far the most common, was associated with divergent *Cx. pipiens* members, including *Cx. quinquefasciatus*, *Cx. pipiens pipiens* form *pipiens* and *Cx. pipiens pipiens* form *molestus*. Four other *w*Pip groups were also found in mosquito populations and all were shared between diverse *Cx. pipiens* members.

**Conclusion:**

This data overall supports the hypothesis that *w*Pip infections, and their allied mitochondria, are associated with regular transfers between *Cx. pipiens* members rather than specific host associations. Overall, this is suggestive of a recent and likely ongoing cytoplasmic introgression through hybridization events across the *Cx. pipiens* complex.

## Background

Symbiotic associations with the intracellular bacterium *Wolbachia* are extremely widespread in insects [1-3]. *Wolbachia* is typically maternally inherited through the egg cytoplasm and has evolved a variety of interactions with its hosts, exerting subtle effects such as manipulation of host reproduction or protection against natural enemies [4-6]. Each of these effects is advantageous to infected females and thus enables *Wolbachia* to spread rapidly through insect populations. These effects are also of ecological and evolutionary importance to the particular host species that is infected, potentially inducing reproductive isolation or driving changes in sexuality [4-6].

Mosquitoes of the *Culex pipiens* complex have long been recognized to exhibit a great variability of effects associated with *Wolbachia*. In this host, *Wolbachia*, known as *w*Pip, is associated with cytoplasmic incompatibility (CI), a sperm-egg incompatibility between infected males and uninfected females, so that infected females have a reproductive advantage [7-9]. Apart from this simple case, CI has been also observed in a number of cases between males and females carrying incompatible *w*Pip strains [9-12]. Five housekeeping genes developed for *Wolbachia* multilocus strain typing (MLST) [13] and the *Wolbachia* surface protein gene *wsp* present no variation among these *w*Pip strains, showing that they delineate a recent and monophyletic clade into the B *Wolbachia* supergroup [14]. The recent examination of fast evolving markers, such as the *ank2* and *pk1* genes encoding proteins with ankyrin (ANK) motifs, revealed the existence of more than 100 genetically distinct *w*Pip strains [9,11,14], belonging to five distinct subclades and further referred as *w*Pip-I to *w*Pip-V groups [14]. Since their molecular characterization, several recent studies have further showed that some *w*Pip strains are mutually incompatible but also that some others, although genetically distinct, are fully compatible [9,11,14]. Meanwhile, *w*Pip has also emerged as a conditional mutualist that protects *Cx. pipiens* against mortality induced by the avian malaria parasite *Plasmodium relictum*[15]. Overall, these studies revealed that *w*Pip is an important associate of most mosquitoes in the *Cx. pipiens* complex potentially driving its evolution.

How *w*Pip infections interact with the genetic structure within the *Cx. pipiens* complex remains still unclear. Mosquitoes in this complex have a global distribution in all temperate and tropical regions, with a recent history of association with human migration [16,17]. The complex encompasses a group of genetically closely related taxa with distinct behavioural and physiological traits that greatly influence their distribution. The most obvious variable traits include larval habitat preference, vertebrate feeding pattern, mating behaviour, gonotrophic development and ability to enter into diapause during the winter [17]. Despite these differences, members of the *Cx. pipiens* complex have controversial taxonomic statuses (including subspecies, forms or biotypes delineations) and, even though they are genetically distinct, they remain difficult to separate morphologically. Four species, or subspecies according to the authors, are frequently recognized as members of the complex: *Cx. pipiens*, *Cx. quinquefasciatus*, *Cx. australicus*, and *Cx. globocoxitus*, as well as two subspecies: *Cx. pipiens pipiens* in Europe and North and South Africa, and *Cx. p. pallens*, in Asia. In addition, two sympatric forms, *pipiens* and *molestus*, are also encountered in *Cx. p. pipiens* in the Northern hemisphere. *Cx. quinquefasciatus*, commonly known as the southern house mosquito, exists all across the tropics and the lower latitudes of temperate regions. *Cx. australicus* and *Cx. globocoxitus* are restricted to Australia and are poorly known. Although the exact taxonomic status of the *Cx. pipiens* members remains controversial, their close evolutionary association has been repeatedly supported by genetic analyses and the relative abundance of hybrids in areas where distributions overlap [18-22]. Remarkably, whilst *w*Pip was never detected in *Cx. australicus* and *Cx. globocoxitus*, infection frequency was near or at fixation in almost all populations of both *Cx. pipiens* and *Cx. quinquefasciatus*[8,23-25]. When first described, any of the five *w*Pip groups were found associated within given infected members of the *Cx. pipiens* complex [14]. However, only a limited number of *Cx. pipiens* and *Cx. quinquefasciatus* laboratory lines have been examined, making this result difficult to interpret. This issue is of special importance since *Wolbachia* may either produce reproductive isolation between host populations infected with different *Wolbachia* strains, or reinforce an existing divergence by selecting for pre-mating isolation mechanisms [4,5]. The presence of closely related host taxa within the *Cx. pipiens* complex is thus a relevant system to test such a hypothesis.

Here we have approached this issue by undertaking an extensive screening for the presence, the diversity and the partitioning of *w*Pip infections in natural populations of the four main members of the *Cx. pipiens* complex (*Cx. quinquefasciatus*, *Cx. p. pipiens* form *pipiens*, *Cx. p. pipiens* form *molestus* and *Cx. p. pallens*), spanning 118 natural populations and 64 laboratory lines. With this aim, we characterized each *w*Pip individual infection using two to five *Wolbachia* markers and identified *Cx. pipiens* members using diagnostic nuclear markers, including microsatellites for a subsample of populations. Since *Wolbachia* and host mitochondria are co-transmitted in egg cytoplasm and therefore are in linkage disequilibrium, we also examined the mitochondrial (mtDNA) diversity through the sequencing of one to three mtDNA markers. Using this approach, we thus attempted to infer the contribution of *Wolbachia* in shaping the genetic diversity within the *Cx. pipiens* complex.

## Methods

### Mosquito collection

We examined mosquitoes from Europe, Asia, Oceania, Africa and America belonging to the four members of the *Cx. pipiens* complex: *Cx. p. pipiens* form *pipiens*, *Cx. p. pipiens* form *molestus*, *Cx. p. pallens* as well as *Cx. quinquefasciatus*, hereinafter respectively referred to as *pipiens*, *molestus*, *pallens* and *quinquefasciatus* (Additional file 1: Table S1). The collection encompasses both natural populations (mostly sampled during the period 1990 to 2012) and isofemale laboratory lines (derived from field specimens collected from 1950 to 2011). Each laboratory line descended from a single female founder and was further considered as a single individual. All specimens were stored in liquid nitrogen or in 70-95% ethanol, at room temperature or in a freezer at −20°C until examined.

### Molecular typing

The *w*Pip infections were genotyped and assigned to one group (*w*Pip-I to *w*Pip-V) using a series of specific PCR-RFLP (restriction fragment length polymorphism) assays based on two ANK *Wolbachia* markers, *ank2* and *pk1* (Additional file 1: Table S2 and Figure S1). *Hinf*I digestion of the *ank2* PCR products allowed discrimination of five alleles (a to e): a (one RFLP fragment: 313 bp), b (217, 195, 98 bp), c (293, 217 bp), d (217, 195 bp) and e (415 bp). *Taq*I digestion of the *pk1* PCR products allowed discrimination of four specific *w*Pip alleles (alleles a and e have the same fragment size): a/e (903, 430 bp), b (669, 665 bp), c (851, 498 bp) and d (497, 251, 107 bp). The *pk1* a and e alleles were next distinguished using a digestion of the *pk1* PCR products with *Pst*I: a (903, 303, 141 bp) and e (903, 430 bp). For a subsample of specimens, three additional *Wolbachia* markers were sequenced: the DNA mismatch repair protein gene *MutL*, the putative secreted protein gene *GP15* (also called *VrlC*) and the regulatory protein gene *RepA* (Additional file 1: Table S2). Four (*ank2*, *MutL*, *GP15* and *RepA*) of these five *Wolbachia* genes were present in one single copy in the *w*Pip(Pel) genome while the fifth (*pk1*) is present in three identical copies (Additional file 1: Table S2; see [14] and [26], for details). A total of 5 *Wolbachia* genes, encompassing 7 distinct loci with a wide distribution along the *w*Pip(Pel) chromosome, were thus examined. None of these genes was amplified from *Wolbachia*-free *Cx. pipiens* lines, which confirmed their *Wolbachia* origin.

DNA was extracted from individual mosquitoes using a CetylTrimethylAmmonium Bromide (CTAB) [27] or phenol/chloroform protocols [28]. All PCR amplification conditions were: 5 min at 94°C, followed by 30–40 cycles of 94°C for 30s, 50°C-58°C for 30s, and 72°C for 1 to 1.5 min depending on the fragment size (detailed on Additional file 1: Table S2). Digestion of PCR products were performed following manufacturer’s instructions. The QIAquick gel extraction kit (QIAGEN, Valencia, CA) was used to purify the PCR products for sequencing. Sequences were obtained directly from purified products on an ABI Prism 3130 sequencer using the BigDye Terminator Kit (Applied Biosystems).

The *Cx. pipiens* mtDNA haplotypes were determined through the sequencing of an 852 bp fragment from the *cytochrome b* (*cytb*) gene (Additional file 1: Table S2). For a subsample of specimens, we also obtained partial sequences of two additional mtDNA markers: the NADH dehydrogenase subunit 2 (*ND2*, 1160 bp) and 5 (*ND5*, 1132 bp) (Additional file 1: Table S2).

The four *Cx. pipiens* taxa were identified using nuclear DNA of specimens. We included in this study some populations available from previous studies for which *Cx. pipiens* taxa were determined using 7 to 12 microsatellite loci and/or sequence variation in an intron of the acetylcholinesterase-2 (*ace-2*) gene (Additional file 1: Table S1). The taxa of new *Cx. pipiens* populations were identified using a combination of the PCR-RFLP on *ace-2*[29,30] and a multiplex-PCR assay based on the flanking region of a microsatellite locus (*CQ11*; see [31] for more details) (Additional file 1: Table S1 and Table S2). Specimens from these new populations were considered as hybrids when they showed a heterozygous genotype at *ace-2* or *CQ11* loci.

### Data analyses

Sequence alignments were carried out using ClustalW [32] and corrected using MEGA [33]. The GBLOCKS program [34] with default parameters was used to remove poorly aligned positions and to obtain unambiguous sequence alignments. The evolutionary model most closely fitting the sequence data was determined using Akaike information criterion with the MEGA program [33]. Phylogenetic analyses were conducted using maximum likelihood (ML) and maximum-parsimony (MP) in MEGA [33]. ML phylogenies were constructed based upon unambiguously aligned sites using the Tajima-Nei model of nucleotide substitution, assuming that nucleotide frequencies deviate substantially from 0.25 [35]. MP phylogenies were constructed using the close-neighbour-interchange method [36]. Bootstrap probabilities were calculated by generating 500 bootstrap replicates. New sequence data were deposited in GenBank (accession numbers KC686688- KC686692).

The microsatellite data was first examined for compliance with Hardy–Weinberg equilibrium, and then pair-wise *F*_ST_ values and their significance were obtained using GENEPOP v4.2 [37]. We assigned specimens to genetic clusters with a maximum likelihood algorithm implemented in the program Structure 2.3.4 [38]. This method combines all the individual multilocus genotypes and separates them into K distinct clusters. We used the admixture model taking into account correlated allele frequencies between populations with 10,000 burn-in steps and 100,000 runs as MCMC (Markov chain Monte Carlo) parameters to assign specimens to genetic clusters. The degree of admixture, alpha, was inferred from the data (if alpha approaches zero most individuals are not admixed). With this parameter set, the geographic origin of each specimen is not considered but the number of clusters (from K = 1 to K = 7) is decided a priori for each run. To assess the consistency of the analysis we performed an exhaustive comparison of 10 runs for each K to assess the robustness of the results. We also performed the method described in Evanno et al. [39] to confirm that the true number of clusters explaining our data was detected.

The significance of non-random associations between *w*Pip/mtDNA, *w*Pip/*Culex pipiens* taxa or mtDNA/*Culex pipiens* taxa was estimated using an exact test procedure (Fisher exact test) implemented in GENEPOP [37].

## Results

### Polymorphism of wPip infections

We assayed for the presence and the diversity of *Wolbachia* in 1935 specimens from 118 natural populations and 64 isofemale laboratory lines (Additional file 1: Table S1). Specific *ank2* and *pk1* PCR assays indicated the occurrence of infection by *Wolbachia* in all the examined specimens, showing that infection is fixed in all *Cx. quinquefasciatus*, *Cx. p. pipiens* form *pipiens*, *Cx. p. pipiens* form *molestus* and *Cx. p. pallens* populations examined here.

**Figure 1:**
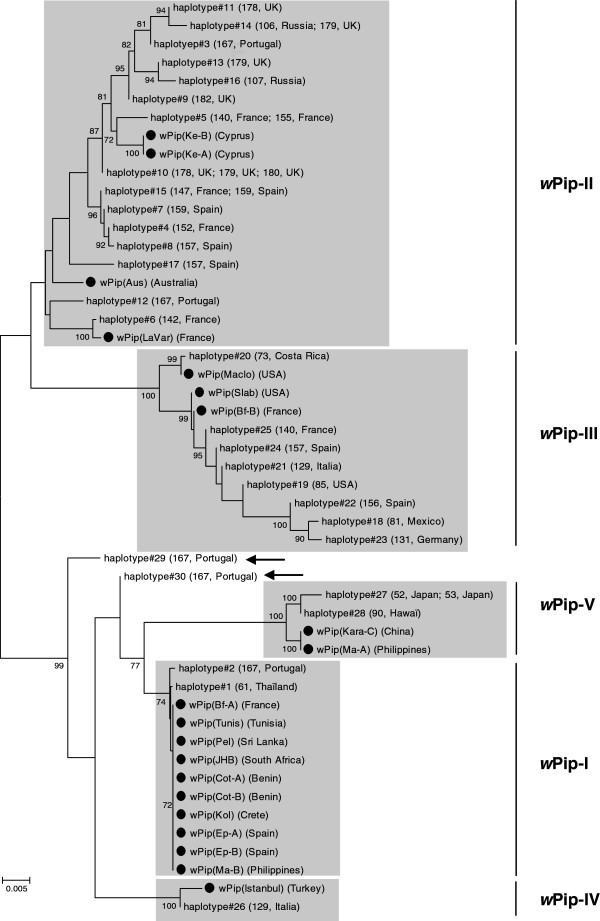
***w*Pip haplotypes phylogeny constructed using Maximum Parsimony method based on concatenated sequences of *ank2*, *pk1*, *MutL*, *GP15 *and *RepA *genes.** Known *w*Pip strains haplotypes are marked by full circles. Arrows show the two non-assigned haplotypes. Numbers on branches indicate percentage bootstrap support (500 replicates). Only bootstrap values > 70 were shown. The scale bar indicates the number of substitutions. Numbers in brackets correspond to locality numbers in Additional file 1: Table S1.

We further used the *ank2* and *pk1* allelic profiles to assign a *w*Pip group to each specimen (Table 1). We found in our collection the five known *ank2* alleles and the five known *pk1* alleles. Tests for intergenic recombination revealed significant linkage disequilibrium (LD) for *ank2* and *pk1* (Fisher exact test, *P* < 10^-5^): alleles at these loci are not randomly associated showing that they are stably co-transmitted within the *w*Pip chromosome. Hence, the *ank2* and *pk1* allelic profiles are congruent and allowed unambiguously attributing the *w*Pip group to 1836 (94.9%) of 1935 specimens (Table 1). For the 99 (5.1%) remaining specimens, *ank2* and *pk1* allelic profiles were not congruent with known haplotypes (Table 1), suggesting that these two loci had undergone recombination. To assign these 99 specimens to a *w*Pip group, three additional *Wolbachia* markers were then sequenced: *MutL, GP15* and *RepA*. Three to thirteen alleles were detected for each marker and led to the identification of 30 new *w*Pip haplotypes from the 99 unassigned specimens (Table 1). Some of these alleles are null alleles: some specimens did not amplify at one of the loci (because of either mutation in the primer sites or gene deletion) showing that their *w*Pip infections were genetically distinct from other known haplotypes. Positive amplifications of other loci (*pk1*, *ank2*) from samples with null alleles at either *MutL, GP15* or *RepA* loci indicate satisfactory DNA template quality in these cases. Phylogenetic tree using *ank2*, *pk1*, *MutL, GP15* and *RepA* concatenated sequences (5624 bp) showed that almost all new *w*Pip haplotypes fall into one of the five *w*Pip groups (Figure 1). Overall, the use of the three additional *Wolbachia* markers allowed the assignment to a *w*Pip group to 96 of the 99 remaining specimens, with only two haplotypes (#29 found in two specimens and #30 in one specimen) remaining not assigned.

**Table 1:** **Allelic profiles of*Wolbachia*genes in the five*w*Pip groups**

***w*Pip group**	***w*Pip Haplotypes**	**n**	***w*Pip Haplotypes**					**Population numbers**
			***ank2***	***pk1***	***GP15***	***MutL***	***RepA***	
*w*Pip-I								
	*w*Pip-I known haplotypes	821	a	a	a	a	a,b	1-45 /57-63/ 66-71/ 74-75/ 77-78/ 105/ 108-114/ 119- 120/ 123-124/ 129/ 146/ 162-165/ 167-168/ 175-176
	#1	1	d	a	a	a	a	61
	#2	1	b	a	ND	ND	ND	167
*w*Pip-II								
	*w*Pip-II known haplotypes	276	e	c	b,e,f	b,c,d	a	64/ 160-107/ 115-117/ 121-122/ 130-132/ 137-145/ 147/ 152-155/ 157/ 159-160/ 165-167/ 179-182
	#3	1	a	c	j*	c	b	167
	#4	1	a	c	f	-	a	152
	#5	2	a	c	b	c	a	140/156
	#6	3	a	c	b	b	a	142
	#7	3	b	c	c	ND	b	147/159
	#8	1	b	c	c	e	b	157
	#9	3	b	c	ND	c	b	182
	#10	9	b	c	ND	ND	b	178/179/180
	#11	4	b	c	h*	c	a	178
	#12	3	b	c	i*	b	b	167
	#13	1	b	c	k*	c	a	179
	#14	7	b	c	h*	c	ND	106/179
	#15	1	b	c	c	-	b	147
	#16	10	b	c	k	c	-	107
	#17	1	c	c	d	-	a	157
*w*Pip-III								
	*w*Pip-III known haplotypes	401	b	b	c	b,e	b	18/ 72-76/ 78-89/ 92-94/ 96-99/ 131/ 134/ 136/ 140/ 144-145/ 148-152/ 154-156/ 158-161/ 165-167/ 179/ 182
	#18	1	a	b	-	-	-	81
	#19	1	c	b	-	-	b	85
	#20	6	d	b	c	b	b	73
	#21	1	e	b	c	-	b	129
	#22	1	e	b	l*	ND	a	156
	#23	1	e	b	ND	ND	ND	131
	#24	1	e	b	f	c	a	157
	#25	1	e	b	c	e	b	140
*w*Pip-IV								
	*w*Pip-IV known haplotype	260	c	d	d	c	a	51/ 90/ 100-104/ 118/ 121/ 125-129/ 131/ 133/ 135/ 169-174
	#26	1	e	d	d	-	a	129
*w*Pip-V								
	*w*Pip-V known haplotype	78	d	e	g	f	a	45-50/ 54/ 65/ 100/ 106
	#27	19	a	e	g	c	a	52/53
	#28	11	b	e	g	a	a	90
undetermined								
	#29	2	b	a	c	c	b	167

New *w*Pip haplotypes are identified by #number. Allelic profiles for *GP15*, *MutL* and *RepA* loci were determined for all new *w*Pip haplotypes and for a subsample of specimens for the known *w*Pip haplotypes. ND, allele not determined (DNA shortage); Dash, null allele (no PCR product); Asterisk, new allele.

Taking into account PCR-RFLP and phylogenetic grouping, we thus found that 823 specimens (42.5%) were infected by the *w*Pip-I group, 326 (16.9%) by *w*Pip-II, 414 (21.4%) by *w*Pip-III, 261 (13.5%) by *w*Pip-IV, 108 (5.6%) by *w*Pip-V and 3 (0.1%) by undetermined *w*Pip groups (Table 1). We found no evidence of specimens infected by more than one *w*Pip group: only one allele per gene was observed for each DNA sample. The subsequent sequencing of *ank2* and *pk1* PCR products obtained from a subsample of specimens confirmed the observed RFLP profiles, with no double peaks (indicative of multi-infection) in electropherograms.

### Geographic distribution of wPip diversity

**Figure 2:**
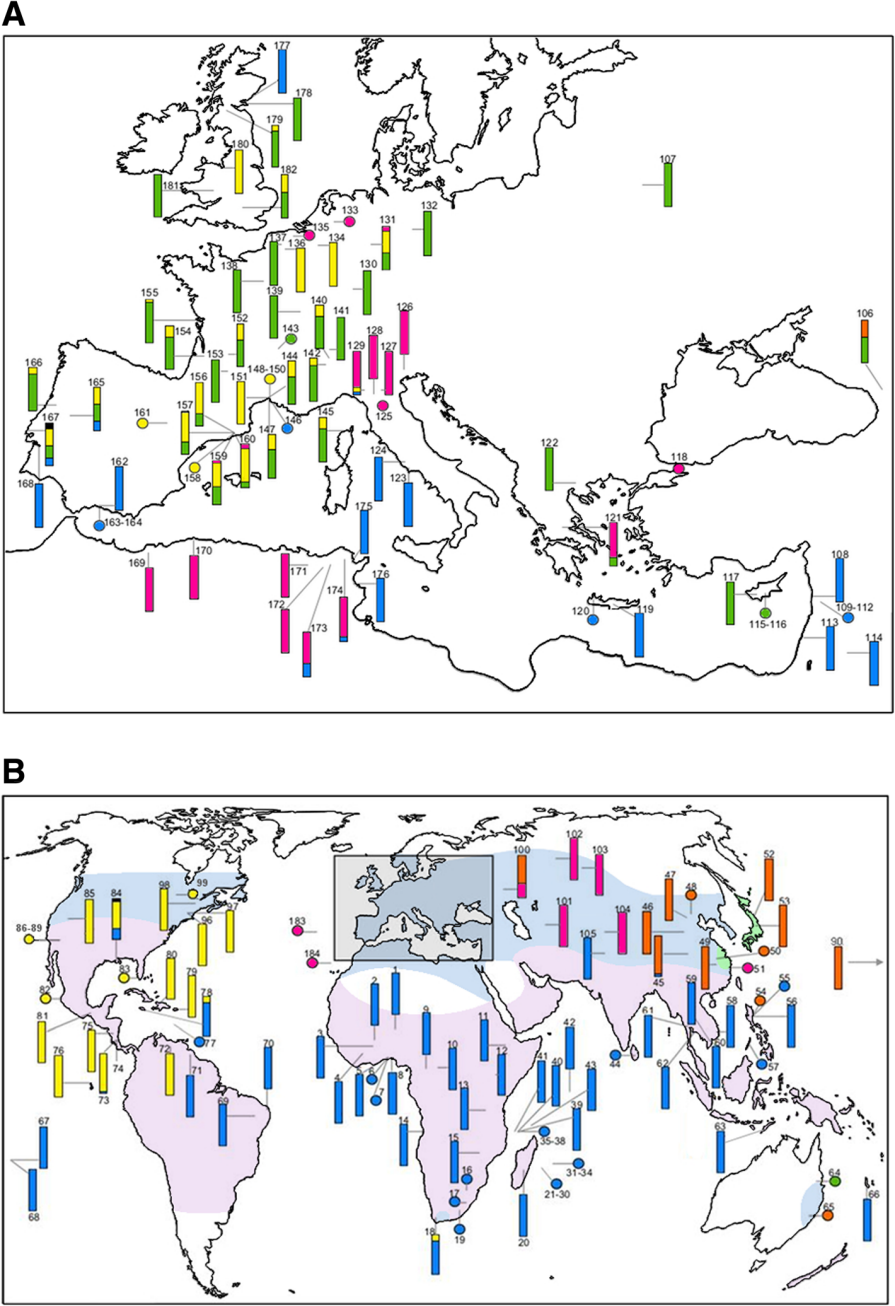
**Geographic distribution of *w*Pip groups in the World (A) and in Europe (B).** Numbers in the maps correspond to locality numbers in Additional file 1: Table S1. Bars and dots represent natural populations and laboratory strains, respectively. The bars show the prevalence of *w*Pip group: blue, *w*Pip-I infection; green, *w*Pip-II; yellow, *w*Pip-III; pink, *w*Pip-IV; orange, *w*Pip-V; black, undetermined group. On Figure 2**A**, *Culex pipiens pipiens* form *pipiens* and *Culex pipiens pipiens* form *molestus* = light blue; *Cx. p. pallens* = light green and *Cx. quinquefasciatus* = light pink (modified after Farajollahi et al. 2011). On Figure 2**B**, both *Cx. p. pipiens* form *pipiens* and *Cx. p. pipiens* form *molestus* are widespread in Europe. Details on sample size, prevalence and *Cx. pipiens* complex taxa are given in Additional file 1: Table S1.

The diversity of *w*Pip showed an important spatial variation over the distribution area of the *Cx. pipiens* complex (Figures 2A, 2B, Additional file 1: Table S1). Two *w*Pip groups were found to dominate wide geographic regions: only *w*Pip-I was found in Sub-Saharan Africa, South America and Southeast Asia, and only *w*Pip-III was observed in North America. The three other groups, *w*Pip-II, *w*Pip-IV and *w*Pip-V, were less common. While *w*Pip-II and *w*Pip-V were confined to Western Europe and Asia, respectively, with very few exceptions, *w*Pip-IV was more disseminated and was sporadically found in Europe, North Africa and Asia. Generally, only one *w*Pip group was found per geographic region indicating a regional homogeneity of *Wolbachia* infections (Figure 2A), but a contrasting picture emerged in Europe where all five *w*Pip groups were found (Figure 2B). Given that Europe is oversampled (709 individuals from 66 populations; Additional file 1: Table S1) relative to other parts of the world, the *w*Pip diversity observed there could be simply a function of a higher degree of sampling effort. However, some regions have been also extensively sampled and still revealed fewer *w*Pip groups (i.e. Africa: 569 individuals from 51 populations but only two *w*Pip groups). Thus, oversampling does not seem to be a likely explanation for the great *w*Pip diversity found in Europe.

In most cases, only one *w*Pip group was detected per population: 88 (74.6%) of the 118 natural populations harboured only one *w*Pip group while, in the remaining 30 (25.4%), two to three *w*Pip groups per population were observed. Twenty-two of the 30 populations were located in Europe and half (18 of 30) harboured *Cx. pipiens* individuals infected either by *w*Pip-II or *w*Pip-III. Aside from the *w*Pip-II/*w*Pip-III mixed populations found in Western Europe, at least five other geographic contact zones between *w*Pip groups exist: in the North (*w*Pip-II/*w*Pip-IV) and in the South of Italy (*w*Pip-I/*w*Pip-IV), in North Africa (*w*Pip-I/*w*Pip-IV), in South America (*w*Pip-I/*w*Pip-III) and Eastern Asia (*w*Pip-I/*w*Pip-V) (Figures 2A, 2B).

### Association of wPip groups with mtDNA and members of the *Cx. Pipiens* complex

To investigate the association between *w*Pip groups and mtDNA variation, we sequenced the *cytb* gene (that have been shown polymorphic in the *Cx. pipiens* complex, see [14]) in a subsample of 184 specimens from 101 *Cx. pipiens* natural populations and 44 isofemale laboratory lines. The *cytb* gene displayed low variability and eleven haplotypes with only ten variable nucleotide positions throughout the 852 bp *cytb* fragment (ca. 99.5% of pairwise identity) were obtained (Additional file 1: Table S3). Pairwise tests revealed significant LD for *w*Pip groups and *cytb* haplotypes (*P* = 10^-5^) with a clear pattern of *cytb* haplotype specificity to *w*Pip groups (Table 2). According to this association and following previous study [14], *cytb* haplotypes were partitioned in five mtDNA groups (mtDNA-1 to 5 associated to *w*Pip-I to V groups, respectively; Table 2).

**Table 2:** **Mitochondrial (*cytb*) haplotypes and partitioning between*w*Pip groups**

**mtDNA group**	**cytb haplotype**	**n**	***w*Pip-I**	***w*Pip-II**	***w*Pip-III**	***w*Pip-IV**	***w*Pip-V**	**Undetermined**
mtDNA-1								
	#1	1	1	0	0	0	0	0
	#2	50	43	0	0	0	7	0
	#3	3	3	0	0	0	0	0
	#4	18	18	0	0	0	0	0
mtDNA-2								
	#5	43	0	39	3	0	0	1
mtDNA-3								
	#6	43	0	1	41	0	0	1
	#7	3	0	0	3	0	0	0
mtDNA-4	#8	5	1	0	0	4	0	0
	#9	16	0	0	0	16	0	0
mtDNA-5								
	#10	1	0	0	0	0	1	0
	#11	1	0	0	0	0	1	0
	Total	184	66	40	47	20	9	2

When analysing the congruence between the five mtDNA and *w*Pip groups, very little incongruence were observed. For instance, although the mtDNA-1 group contains 65 *w*Pip-I-infected specimens, seven specimens infected with *w*Pip-V were also found in this group (Table 2). A very similar pattern was found for five other specimens: one *w*Pip-I-infected specimen was found in the mtDNA-4 group (otherwise associated to 20 *w*Pip-IV-infected specimens), one *w*Pip-II-infected specimen in the mtDNA-3 group (associated to 44 *w*Pip-III-infected specimens), and 3 *w*Pip-III-infected specimens in the mtDNA-2 group (associated to 39 *w*Pip-II-infected specimens). To improve our understanding of this incongruence, two additional mtDNA genes (*ND2* and *ND5*) were sequenced and phylogenetic analyses were conducted using *cytb*, *ND2* and *ND5* concatenated genes (2549 bp; Additional file 1: Figure S2). Only the seven *w*Pip-V-infected specimens found associated with the mtDNA-1 group on the basis of *cytb* sequence were analysed since not enough DNA was available from the five remaining specimens. These analyses also included the two unassigned *w*Pip haplotypes (#29 and #30) described above. The resulting phylogenetic tree does not group the seven incongruent specimens infected by *w*Pip-V within the extended mtDNA-1 group, and they can not be assigned to a specific mtDNA group. These results suggest that the primary *w*Pip-mtDNA incongruence observed in these specimens is likely due to low polymorphism in the mitochondrial gene studied here (see Additional file 1: Table S3). The two unassigned *w*Pip haplotypes, #29 and #30, were clearly associated with mtDNA-3 and mtDNA-2, respectively. The strong association between mtDNA and *w*Pip infection presented above indicates that these two haplotypes could be assigned with a low risk of error to *w*Pip-II and *w*Pip-III groups.

To investigate the association of *w*Pip groups with taxa within the *Cx. pipiens* complex, we examined the partitioning of *w*Pip groups among 409 *Cx. pipiens* specimens assigned to one of four *Cx. pipiens* complex taxa: *quinquefasciatus* (n = 201, 49.1%), *pipiens* (n = 118, 28.8%), *molestus* (n = 64, 15.7%), and *pallens* (n = 20, 4.9%) as well as a few hybrids (n = 6, 1.5%) (Additional file 1: Table S1). The observed geographic distribution of our specimens was very similar to the known distribution of the *Cx*. *pipiens* taxa [17,30] with *quinquefasciatus* widely present in tropical areas, *pipiens* and *molestus* in Europe, North Africa, Middle East and North America, and *pallens* confined to East Asia (Figures 2A, 2B). The association between *Cx. pipiens* taxa and *w*Pip groups was highly significant (Fisher exact test, *P* < 10^-4^), as shown with *quinquefasciatus* that appeared mainly infected with the *w*Pip-I group (133 of 201 individuals), *pipiens* with *w*Pip-II (55 of 118) and *pallens* with *w*Pip-V (19 of 20) (Table 3). However, the pattern is less clear for *molestus* that showed a more balanced prevalence of *w*Pip groups with *w*Pip-I, *w*Pip-II and *w*Pip-III more or less equally prevalent in *molestus* (each infecting 15 to 20 of 64 individuals). Overall, the association *w*Pip/*Culex pipiens* taxa is thus far from exclusive since no *w*Pip group was unique to a particular *Cx. pipiens* member: each *Cx. pipiens* taxa harbours two to five different *w*Pip groups. For instance, *pipiens* harbours infections of all five *w*Pip groups and *quinquefasciatus* of three *w*Pip groups (*w*Pip-I, *w*Pip-III and *w*Pip-V).

**Table 3:** **Partitioning of*w*Pip groups among*Cx. pipiens*taxa**

			***Culex pipiens *taxa**			
***w*Pip infection**	**n**	***quinquefasciatus***	***pipiens***	***molestus***	***pallens***	**Hybrids**
*w*Pip-I	171	133	16	20	0	2
*w*Pip-II	62	0	55	7	0	0
*w*Pip-III	96	46	30	20	0	0
*w*Pip-IV	31	0	15	15	1	0
*w*Pip-V	47	21	2	1	19	4
Undetermined	2	0	1	1	0	0

**Figure 3:**
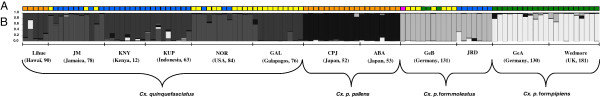
**Comparison of *w*Pip groups (A) with *Cx. pipiens *genetic clusters revealed by Bayesian analysis using microsatellites loci (B).** Each of the 113 individuals included in the analysis is represented by a vertical line, partitioned into five squares assigned different colours (blue, *w*Pip-I infection; green, *w*Pip-II; yellow, *w*Pip-III; pink, *w*Pip-IV; orange, *w*Pip-V) **(A)** and segments of different colours that represent the individual’s probability of belonging to one of the four genetic clusters (black, *pallens*; medium and dark grey, *quinquefasciatus*; soft grey, *molestus*; white, *pipiens*) **(B)**. Specimens were grouped by location (bracketed), and the indicated population numbers are the same as in Additional file 1: Table S1.

Alternatively, the heterogeneity of *w*Pip infections among *Cx. pipiens* members could be based on potential artefacts from molecular typing: for some samples, taxa identification was done through the characterization of two nuclear markers, *ace-2* and *CQ11*, an approach that could fail to detect hybrid individuals. To address this potential bias we refined our analysis by restricting our analyses to a subsample of 113 specimens from 12 populations (encompassing populations of *quinquefasciatus*: populations 12, 63, 76, 78, 84 and 90 in Table S1, *pipiens*: 130 and 181, *molestus*: 114 and 131, *pallens*: 52 and 53) that were typed with microsatellite loci. Although these mosquito populations were originally typed using 7–12 microsatellite loci, the loci used were however different depending on what *Cx. pipiens* taxa and studies they were for. Further analyses were thus conducted using four microsatellite loci (GT4, CxpGT12, GT46 and CQ26; [40,41]) for which genotypes were available for all specimens. None of these loci had significant heterozygote deficits/excess in the examined populations, fitting with Hardy–Weinberg assumptions. A pair-wise *F*_ST_ comparison revealed significant differentiation between mosquito populations belonging to different taxa in all cases (*F*_ST_ values ranged from 0.165 to 0.660; Additional file 1: Table S4), while within taxa the *F*_ST_ values ranged from only 0.012 to 0.251 and were in some cases not different from zero (Additional file 1: Table S4), indicating that gene flow is more important within than between *Cx. pipiens* taxa. The result of the mosquito genetic structure analysis further separates the individuals into five distinct clusters analogous to the *Culex* taxa assignation (Figure 3). Indeed, the clustering separate *pallens*, *pipiens*, *molestus*, *quinquefasciatus* from America and *quinquefasciatus* from Africa and Asia. This clustering indicates a low hybrid rate (alpha ≈ 0.03) indicating that most individuals are not admixed and come from a cluster (Figure 3). The assignment of individuals using microsatellite data was in agreement with our primary assignment, although it separates *quinquefasciatus* populations in two distinct clusters depending on their geographic origins. Same as above, the association between *w*Pip groups and *Cx. pipiens* members was still significant (Fisher exact test, *P* < 10^-4^) and also confirmed that this association is not exclusive since at least four *w*Pip groups are shared by different *Cx. pipiens* members (Figure 3). The presence of shared *w*Pip groups between *Cx. pipiens* members thus suggests that *w*Pip infections undergo repeated transfers between *quinquefasciatus*, *pipiens*, *molestus* and *pallens*.

Finally, the association between mtDNA and *Cx. pipiens* members was assayed in 166 individuals (Table 4). As observed with *w*Pip groups, there was a significant association between mtDNA and *Cx. pipiens* members (Fisher exact test, *P* < 10^-4^), but not exclusive: although mtDNA-1 group was common in *quinquefasciatus* (59 of 72 individuals) and mtDNA-2 group was common in *pipiens* (35 of 67), no mtDNA group was specific to a particular *Cx. pipiens* member (Table 4). As could be expected due to LD between *w*Pip and mtDNA, the mtDNA distribution thus mirrored the *w*Pip distribution across the *Cx. pipiens* complex, showing that they form together a single cytoplasmic unit. Overall, these results show that *w*Pip and mtDNA share a joint evolutionary history, subtly different to the one of nuclear DNA and thus to the evolutionary histories of *Cx. pipiens* members.

**Table 4:** **Partitioning of*cytb*haplotypes between*Cx. pipiens*taxa. Specimens with a hybrid signature were not included in these analyses**

			***Culex pipiens*taxa**			
**mtDNA_group**	***cytb*haplotypes**	**n**	***quinquefasciatus***	***pipiens***	***molestus***	***pallens***
mtDNA_1	#1	1	1	0	0	0
	#2	48	43	4	0	1
	#3	18	10	3	5	0
	#4	5	5	0	0	0
mtDNA_2	#5	38	0	35	3	0
mtDNA_3	#6	35	10	19	6	0
	#7	2	1	1	0	0
mtDNA_4	#8	5	0	1	4	0
	#9	12	0	4	7	1
mtDNA_5	#10	1	1	0	0	0
	#11	1	1	0	0	0

## Discussion

We sampled four evolutionarily closely related mosquito taxa within the *Cx. pipiens* complex for *Wolbachia* infection study. We further specifically tested for host-specific associations by characterizing *Wolbachia* and mtDNA haplotypes using multilocus typing schemes.

The observed prevalence of *w*Pip infection was 100% in all *Cx. quinquefasciatus*, *Cx. p. pipiens* form *pipiens*, *Cx. p. pipiens* form *molestus* and *Cx. p. pallens* populations, as usually recorded for these *Cx. pipiens* taxa [8,25,42]. The genotyping of *w*Pip strains using the two ANK genes *ank2* and *pk1*confirmed the presence of five distinct *w*Pip groups as observed in a previous study [14] showing that *ank2* and *pk1* genes are suitable to assign *w*Pip groups. However, by using additional *w*Pip markers we detected greater diversity including new and recombinant haplotypes indicating higher *w*Pip diversity than previously thought. Overall, our survey of infection diversity is therefore likely to seriously underestimate the true figure of *w*Pip diversity that shows a rapid diversification in their natural host *Cx. pipiens*.

The distribution of *w*Pip groups appeared spatially structured, well exemplified by European *Cx. pipiens* populations that harbour the highest *w*Pip diversity across all examined geographic regions. This suggests that the *w*Pip ancestor may have initially spread in European populations where it evolved in five divergent groups, and that newly emerged *w*Pip groups have only secondarily expanded outside Europe. Human migrations have also probably enhanced this process by expanding the geographic range of diverse *Cx. pipiens* members. For instance, both *Cx. pipiens* and *Cx. quinquefasciatus* were recently introduced into the Americas and Australia [16,17,19]. In the Americas, the presence of two *w*Pip groups indicates that at least two separate introductions have occurred: one probably from Europe introduced *w*Pip-III-infected *Cx. pipiens* to North America, the other from tropical Africa or South Asia introduced *w*Pip-I-infected *Cx. quinquefasciatus* to Americas. In Australia, independent introduction events may also explain the presence of *w*Pip-II and *w*Pip-V, otherwise mainly found in Western Europe and Asia. That American and Australian populations came from multiple and independent colonization events from Europe, Africa and Asia is also well supported by the examination of *Cx. pipiens* microsatellites [18,19]. Overall, the *w*Pip distribution is likely to result from ancient and recent imprints, underlining the importance of historical contingencies in the population structure of infections.

Remarkably, the mtDNA variation in the *Cx. pipiens* complex mirrored precisely the *w*Pip variation, showing a great evidence for indirect selection arising from linkage disequilibrium with infections. mtDNA diversity was extremely reduced over the distribution area of *Cx. pipiens* members, which is likely to be a consequence of cytoplasmic hitchhiking driven by the recent invasion of the *w*Pip ancestor, as pointed by previous studies [10,14,24]. The mtDNA of *Cx. pipiens* individuals infected by different *w*Pip groups have further evolved to be distinct, showing that these two cytoplasmic elements have experienced a recent joint evolutionary history. As a result, *w*Pip confounds the inference of *Cx. pipiens* evolutionary history from mtDNA data, as often observed in other *Wolbachia*-infected species [43,44].

Because of vertical transmission, one should expect that persistence of *w*Pip infection in the *Cx. pipiens* complex over long periods of time should result in diversification of *Wolbachia* alongside the host (co-cladogenesis). However, we did not observe this pattern: similar *w*Pip groups and mtDNA haplotypes are found in different taxa and no cytoplasm type is specific to a given *Cx. pipiens* taxa. We rather showed that the cytoplasmic diversity tends to be homogenized across the four members of the *Cx. pipiens* complex we examined. Collectively, the data suggest a recent, and possibly still ongoing, cytoplasmic exchange between *Cx. pipiens* taxa.

Two non-exclusive processes can explain why different *Cx. pipiens* taxa share similar cytoplasms. The first process is the relatively recent emergence of some *Cx. pipiens* taxa. The *pipiens* and *molestus* forms are very closely related and several scenarios place their divergence at 10,000 years ago [18], which is slightly more recent than the supposed emergence of *w*Pip groups (estimated at ca 20,000 years ago; [14]). Assuming that *w*Pip infections were initially present in the *pipiens*-*molestus* ancestor could explain why many shared *w*Pip groups were found in both *Cx. pipiens* taxa. The second process that could lead the homogenization of cytoplasmic diversity is linked to hybridization events within the complex. Although each member of the *Cx. pipiens* complex has an unique genetic signature, their genetic independence is not absolute since occasional inter-taxa hybridization may occur [16, 18, 22, this study]. Hybridization may transfer *w*Pip infections and associated mtDNA from taxa to taxa (i.e. cytoplasmic introgression) [43]. This pattern fits well with the case of *Cx. quinquefasciatus*. This taxon has emerged long before the *pipiens-molestus* divergence [18], and before the initial *w*Pip infection in European populations. It is thus likely that *w*Pip has secondarily spread from *Cx. pipiens* to *Cx. quinquefasciatus* following cytoplasmic introgression since recurring hybridization occurs where their distribution overlaps [16-18]. A similar process could also explain the presence of two different *w*Pip groups in *Cx. p. pallens*: this latter taxa is thought to be the result of unidirectional hybridization between *Cx. p. pipiens* females and *Cx. quinquefasciatus* males [45], a pattern that explains why *Cx. p. pipiens* and *Cx. p. pallens* share two *w*Pip groups. Cytoplasmic introgression is likely still underway in the *Cx. pipiens* complex: hybrids between *pipiens* and *molestus* forms have been documented in North America and in southern Europe [18,22] as well as between *Cx. quinquefasciatus* and *Cx. p. pallens* in Asia [45], and *w*Pip infections may commonly flow from either side into the hybrid zone.

## Conclusions

It is now clear that *Wolbachia* impacts the *Cx. pipiens* diversity in different ways. The *w*Pip distribution presented here showed a well-structured picture, and underlines a critical example of cytoplasmic introgression through sibling taxa. This situation is actually similar to adaptive introgression of beneficial alleles. Previous studies examining loci involved in insecticide resistance concluded that resistant alleles are undergoing frequent expansion across the complex through hybridization between *Cx. pipiens* members [46,47]. Survey of *Cx. pipiens* neutral loci, however, show that recombination may break the connection between selected and neutral loci very quickly, maintaining the genetic differentiation between taxa and allowing the inter-taxa expansion of selected genes [19]. Hybridization can weakly impact the global flow of nuclear genes but serves as a powerful mechanism of rapid adaptation for insect populations through the penetration of useful adaptive alleles. The *w*Pip infections seem to operate in a similar manner as insecticide resistance genes in the *Cx. pipiens* members, suggesting that introgression has a crucial role in the dispersal of *w*Pip infections. Of note, however, in the case of *Wolbachia*, infection spread may have a negative impact on population dynamics of mosquito hosts since it could drive deleterious mitotypes to fixation through cytoplasmic hitchhiking. It remains to be seen what role, if any, CI between incompatible *w*Pip strains play in divergence between different geographic populations within the *Cx. pipiens* complex.

## Competing interests

The authors declare that they have no competing interests.

## Authors’ contributions

Conceived and designed the experiments: ED, CMA, MW and OD. Performed the experiments: ED, CMA, OD, P Makoundou, SU, DMF, EVS. Analyzed the data: ED, P Milesi and OD. Wrote the paper: ED, MW and OD. All authors read and approved the final manuscript.

## Supplementary Material

Additional file 1: Table S1 Detailed results of the screen of *Culex pipiens* populations. LL, laboratory lines; ND, not determined. **Table S2.** List of primers and gene features. **Table S3.** Nucleotide polymorphism in the *cytb*, *ND2* and *ND5* mitochondrial genes. Only polymorphic sites are represented and a dash indicates similarity with the top sequence. Position expressed in nucleotides bases on the complete mitochondrial sequence of Pel *Culex pipiens* line (Klasson *et al.* 2008). **Table S4.** Pair-wise FST values among mosquito populations (*quinquefasciatus*: populations 78, 12, 63, 84, 76 and 90 in **Table S1**, *pallens*: 52 and 53, *molestus*: 131 and 114, *pipiens*: 130 and 181). *, significant *FST* values after Bonferroni correction. **Figure S1.** Identification of *ank2* and *pk1* allelic profiles. (A) *Hinf*I digestion of the *ank2* PCR products allowed discrimination of five alleles (a to e): a (one RFLP fragment: 313 bp), b (217, 195, 98 bp), c (293, 217 bp), d (217, 195 bp) and e (415 bp). (B) *Taq*I digestion of the *pk1* PCR products allowed discrimination of four specific *w*Pip alleles (alleles a and e have the same fragment sizes): a/e (903, 430 bp), b (669, 665 bp), c (851, 498 bp) and d (497, 251, 107 bp). (C) *PstI* digestion of the *pk1* PCR products allowed discrimination alleles a (903, 303, 141 bp) and e (903, 430 bp). **Figure S2.***mtDNA* phylogeny constructed using Maximum likelihood method based on concatenated sequences of *cytb, ND2* and *ND5* genes. mtDNA haplotypes originally described by Atyame *et al*.(2011b) are marked by full circles. Triangles show the seven specimens presenting incongruences between *w*Pip infection and mtDNA haplotypes. Numbers on branches indicate percentage bootstrap support for major branches (500 replicates). The scale bar indicates the number of substitutions. Click here for file
