# Supplementary material for: Population structure of Wolbachia and cytoplasmic introgression in a complex of mosquito species
Source: BMC Evol Biol. 2013 Sep 3;13:181. doi: 10.1186/1471-2148-13-181 (PMC3846486; doi:10.1186/1471-2148-13-181)
Supplement: Additional file 1: Table S1 — Detailed results of the screen of Culex pipiens populations. LL, laboratory lines; ND, not determined. Table S2. List of primers and gene features. Table S3. Nucleotide polymorphism in the cytb, ND2 and ND5 mitochondrial genes. Only polymorphic sites are represented and a dash indicates similarity with the top sequence. Position expressed in nucleotides bases on the complete mitochondrial sequence of Pel Culex pipiens line (Klasson et al. 2008). Table S4. Pair-wise FST values among mosquito populations (quinquefasciatus: populations 78, 12, 63, 84, 76 and 90 in Table S1, pallens: 52 and 53, molestus: 131 and 114, pipiens: 130 and 181). *, significant FST values after Bonferroni correction. Figure S1. Identification of ank2 and pk1 allelic profiles. (A) HinfI digestion of the ank2 PCR products allowed discrimination of five alleles (a to e): a (one RFLP fragment: 313 bp), b (217, 195, 98 bp), c (293, 217 bp), d (217, 195 bp) and e (415 bp). (B) TaqI digestion of the pk1 PCR products allowed discrimination of four specific wPip alleles (alleles a and e have the same fragment sizes): a/e (903, 430 bp), b (669, 665 bp), c (851, 498 bp) and d (497, 251, 107 bp). (C) PstI digestion of the pk1 PCR products allowed discrimination alleles a (903, 303, 141 bp) and e (903, 430 bp). Figure S2.mtDNA phylogeny constructed using Maximum likelihood method based on concatenated sequences of cytb, ND2 and ND5 genes. mtDNA haplotypes originally described by Atyame et al.(2011b) are marked by full circles. Triangles show the seven specimens presenting incongruences between wPip infection and mtDNA haplotypes. Numbers on branches indicate percentage bootstrap support for major branches (500 replicates). The scale bar indicates the number of substitutions. [file 1471-2148-13-181-S1.pdf]

Supplementary materials

Population structure of *Wolbachia* and cytoplasmic introgression in a complex of mosquito species

Emilie Dumas, Célestine M. Atyame, Pascal Milesi, Dina M. Fonseca, Elena V. Shaikevich, Sandra Unal, Patrick Makoundou, Mylène Weill and Olivier Duron

Populations (region, country, name)	Map	Collection year	n	Frequency of *Wolbachia* infections (number of specimens)						Frequency of *Cx. pipiens* taxa (number of examined specimens)					*mitotype (cytb)*	
				*w*Pip(I)	*w*Pip(II)	*w*Pip(III)	*w*Pip(IV)	*w*Pip(V)	Undetermi ned	*quinque- -fasciatus*	*pipiens*	*molestu s*	*pallens*	*hybrides*	mtDNA haplotype	References
Africa																
Niger																
Niamey (LL)	1	1990	18	1.0(18)	_	_	_	_	_	1.0(3)	_	_	_	_	*#2 (1)*	This study
Burkina Faso																
Ouagadougou	2	1997	23	1.0(23)	_	_	_	_	_	1.0(2)	_	_	_	_	*#2 (1)*	This study
Bobo Dioulassoba	3	2011	15	1.0(15)	_	_	_	_	_	1.0(4)	_	_	_	_	*#2 (1)*	This study
Cote d'Ivoire																
Bouake	4	1986	4	1.0(4)	_	_	_	_	_	1.0(1)	_	_	_	_	*#2 (1)*	Magnin et al. 1988
Benin																
Cotonou	5	2003	26	1.0(26)	_	_	_	_	_	1.0(3)	_	_	_	_	*#2 (1)*	Duron et al. 2007
Cotonou-A (LL)	6	2005	1	1.0(1)	_	_	_	_	_	1.0(1)	_	_	_	_	*#2 (1)*	Duron et al. 2007
Cotonou-B (LL)	7	2005	1	1.0(1)	_	_	_	_	_	1.0(1)	_	_	_	_	*#2 (1)*	Duron et al. 2007
Ouidah	8	2011	23	1.0(23)	_	_	_	_	_	1.0(3)	_	_	_	_	*#2 (1)*	This study
Cameroon																
Yaoundé	9	2008	22	1.0(22)	_	_	_	_	_	1.0(3)	_	_	_	_	*#2 (1)*	This study
Central African Republic																
Bangui	10	2011	21	1.0(21)	_	_	_	_	_	1.0(3)	_	_	_	_	*#2 (1)*	This study
Ethiopia																
Ethiopia	11	unknown	11	1.0(11)	_	_	_	_	_	1.0(2)	_	_	_	_	*#2 (1)*	This study
Kenya																
KNY	12	1999	8	1.0(8)	_	_	_	_	_	1.0(8)	_	_	_	_	*#2 (1)*	Fonseca et al. 2006
Tanzania																
Arusha	13	2011	22	1.0(22)	_	_	_	_	_	1.0(3)	_	_	_	_	*#2 (1)*	This study
Angola																
Lobito	14	2011	24	1.0(24)	_	_	_	_	_	1.0(3)	_	_	_	_	*#2 (1)*	This study
Zimbabwe																
Harare	15	2001	17	1.0(17)	_	_	_	_	_	1.0(3)	_	_	_	_	*#2 (1)*	Weill et al. 2003

South Africa																	
	JHB (LL)	16	2001	1	1.0(1)	_	_	_	_	_	ND	ND	ND	ND	ND	*ND*	Salzberg et al. 2009
	Bed (LL)	17	1993	1	1.0(1)	_	_	_	_	_	1.0(1)	_	_	_	_	*#2 (1)*	This study
	Wellington	18	2011	5	0,8(4)	_	0,2(1)	_	_	_	0.5(2)	0.5(1)	_	_	_	*#2 (1), #6 (1)*	This study
	BSQ	19	1993	3	1.0(3)	_	_	_	_	_	1.0(2)	_	_	_	_	*#2 (1)*	Weill et al. 2003
Madagascar																	
	Mada	20	2011	6	1.0(6)	_	_	_	_	_	1.0(1)	_	_	_	_	*ND*	This study
Reunion island																	
	Pie-11 (LL)	21	2007	1	1.0(1)	_	_	_	_	_	1.0(1)	_	_	_	_	*#4 (1)*	Atyame et al. 2010
	Su-132 (LL)	22	2007	1	1.0(1)	_	_	_	_	_	1.0(1)	_	_	_	_	*#4 (1)*	Atyame et al. 2010
	Leu-132 (LL)	23	2007	1	1.0(1)	_	_	_	_	_	1.0(1)	_	_	_	_	*#3 (1)*	Atyame et al. 2010
	Leu-118 (LL)	24	2007	1	1.0(1)	_	_	_	_	_	1.0(1)	_	_	_	_	*#3 (1)*	Atyame et al. 2010
	Su-118 (LL)	25	2007	1	1.0(1)	_	_	_	_	_	1.0(1)	_	_	_	_	*#4 (1)*	Atyame et al. 2010
	Leu-58 (LL)	26	2007	1	1.0(1)	_	_	_	_	_	1.0(1)	_	_	_	_	*#4 (1)*	Atyame et al. 2010
	Pie-58 (LL)	27	2007	1	1.0(1)	_	_	_	_	_	1.0(1)	_	_	_	_	*#3 (1)*	Atyame et al. 2010
	Su-122 (LL)	28	2007	1	1.0(1)	_	_	_	_	_	1.0(1)	_	_	_	_	*#3 (1)*	Atyame et al. 2010
	Leu-122 (LL)	29	2007	1	1.0(1)	_	_	_	_	_	1.0(1)	_	_	_	_	*#3 (1)*	Atyame et al. 2010
	Su-133 (LL)	30	2007	1	1.0(1)	_	_	_	_	_	1.0(1)	_	_	_	_	*#4 (1)*	Atyame et al. 2010
Mauritius																	
	Maurice (LL)	31	2010	1	1.0(1)	_	_	_	_	_	1.0(1)	_	_	_	_	*ND*	This study
	Maurice-4 (LL)	32	2010	1	1.0(1)	_	_	_	_	_	1.0(1)	_	_	_	_	*ND*	This study
	Maurice-5 (LL)	33	2010	1	1.0(1)	_	_	_	_	_	1.0(1)	_	_	_	_	*ND*	This study
	Maurice-7 (LL)	34	2010	1	1.0(1)	_	_	_	_	_	1.0(1)	_	_	_	_	*ND*	This study
Mayotte																	
	Mayotte (LL)	35	2010	1	1.0(1)	_	_	_	_	_	1.0(1)	_	_	_	_	*ND*	This study
	Mayotte-5 (LL)	36	2010	1	1.0(1)	_	_	_	_	_	1.0(1)	_	_	_	_	*ND*	This study
	Mayotte-6 (LL)	37	2010	1	1.0(1)	_	_	_	_	_	1.0(1)	_	_	_	_	*ND*	This study
	Mayotte-17 (LL)	38	2010	1	1.0(1)	_	_	_	_	_	1.0(1)	_	_	_	_	*ND*	This study
	M'tsamoudou	39	2011	24	1.0(24)	_	_	_	_	_	1.0(3)	_	_	_	_	*#2 (1)*	This study
	Acoua	40	2011	23	1.0(23)	_	_	_	_	_	1.0(3)	_	_	_	_	*#2 (1)*	This study
Comoros																	
	Anjouan	41	2011	23	1.0(23)	_	_	_	_	_	1.0(3)	_	_	_	_	*#2 (1)*	This study

	Glorios Island																	
		Grandes Glorieuses	42	2011	23	1.0(23)	_	_	_	_	_	1.0(3)	_	_	_	_	*#2 (1)*	This study
	Seychelles																	
		Mahé	43	2011	23	1.0(23)	_	_	_	_	_	1.0(3)	_	_	_	_	*#2 (1)*	This study
	Algeria																	
		Tafna	169	2006	5	_	_	_	1.0(5)	_	_	ND	ND	ND	ND	ND	*ND*	This study
		Harash	170	2006	24	_	_	_	1.0(24)	_	_	ND	ND	ND	ND	ND	*#9 (1)*	This study
		Constantine	171	2006	5	_	_	_	1.0(5)	_	_	ND	ND	ND	ND	ND	*ND*	This study
		Souk Ahras	172	2008	30	_	_	_	1.0(30)	_	_	ND	ND	ND	ND	ND	*ND*	This study
	Tunisia																	
		Ain Tounga	173	2009	31	0.3(9)	_	_	0.7(22)	_	_	_	1.0(2)	_	_	_	*ND*	This study
		El manar	174	2009	45	0.1(5)	_	_	0.9(40)	_	_	ND	ND	ND	ND	ND	*ND*	This study
		Sokra	175	2008	19	1.0(19)	_	_	_	_	_	ND	ND	ND	ND	ND	*ND*	This study
		Ayed	176	2005	24	1.0(24)	_	_	_	_	_	ND	ND	ND	ND	ND	*ND*	This study
Middle East																		
	Lebanon																	
		Beyrouth	108	2006	22	1.0(22)	_	_	_	_	_	_	_	1.0(3)	_	_	*#3 (1)*	This study
		Liban-1 (LL)	109	2010	1	1.0(1)	_	_	_	_	_	ND	ND	ND	ND	ND	*ND*	This study
		Liban-2 (LL)	110	2010	1	1.0(1)	_	_	_	_	_	ND	ND	ND	ND	ND	*ND*	This study
		Liban-3 (LL)	111	2010	1	1.0(1)	_	_	_	_	_	ND	ND	ND	ND	ND	*ND*	This study
		Liban-4 (LL)	112	2010	1	1.0(1)	_	_	_	_	_	ND	ND	ND	ND	ND	*ND*	This study
	Pakistan																	
		Lahore	105	1988	6	1.0(6)	_	_	_	_	_	1.0(3)	_	_	_	_	*#2 (1)*	Beyssat-Arnaouty et al. 1989
	Israel																	
		Yatouch	113	2010	27	1.0(27)	_	_	_	_	_	_	1.0(3)	_	_	_	*#3 (1)*	This study
	Jordan																	
		JRD	114	1999	7	1.0(7)	_	_	_	_	_	_	_	1.0(7)	_	_	*ND*	Fonseca et al. 2006
	Turkey																*#8 (1)*	
		Istanbul (LL)	118	2003	1	_	_	_	1.0(1)	_	_	_	_	1.0(1)	_	_	*#8 (1)*	Duron et al. 2005
Asia																		
	Sri Lanka																	
		Pel (LL)	44	1984	1	1.0(1)	_	_	_	_	_	ND	ND	ND	ND	ND	*#1 (1)*	Klasson et al. 2008

	China																	
		Zhuchang	45	2003	16	0,1(1)	_	_	_	0,9(15)	_	_	0.3(1)	_	_	0.7(2)	*#2 (2)*	This study
		BJBJT	46	2003	24	_	_	_	_	1.0(24)	_	0.7(2)	_	_	_	0.3(1)	*#2 (1)*	Duron et al. 2005
		Taïqiu	47	2003	13	_	_	_	_	1.0(13)	_	0.67(2)	_	_	_	0.33(1)	*#2 (1)*	This study
		Beijing (LL)	48	2003	1	_	_	_	_	1.0(1)	_	1.0(1)	_	_	_	_	*#2 (1)*	Qiao & Raymond 1995
		Karaoke	49	2003	16	_	_	_	_	1.0(16)	_	1.0(3)	_	_	_	_	*#2 (1)*	Duron et al. 2005
		Kara-C (LL)	50	2003	1	_	_	_	_	1.0(1)	_	1.0(1)	_	_	_	_	*#10 1)*	Duron et al. 2005
		Hang Zhou (LL)	51	unknown	1	_	_	_	1.0(1)	_	_	_	_	_	1.0(1)	_	*#9 1)*	This study
	Japan																	
		CPJ	52	2001	11	_	_	_	_	1.0(11)		_	_	_	1.0(11)	_	*ND*	Fonseca et al. 2009
		ABA	53	2004	8	_	_	_	_	1.0(8)		_	_	_	1.0(8)	_	*ND*	Fonseca et al. 2009
	Philippines																	
		Manille-A (LL)	54	2003	1	_	_	_	_	1.0(1)	_	1.0(1)	_	_	_	_	*#11 1)*	Duron et al. 2006
		Manille-B (LL)	55	2003	1	1.0(1)	_	_	_	_	_	1.0(1)	_	_	_	_	*#2 (1)*	Duron et al. 2006
		Manille	56	2003	24	1.0(24)	_	_	_	_	_	1.0(3)	_	_	_	_	*#2 (1)*	Duron et al. 2005
		Pallawan (LL)	57	2003	1	1.0(1)	_	_	_	_	_	1.0(1)	_	_	_	_	*#2 (1)*	Duron et al. 2005
	Vietnam																	
		Nha Trang	58	1995	9	1.0(9)	_	_	_	_	_	1.0(3)	_	_	_	_	*#2 (1)*	Pasteur et al. 2001
		Hot Toc	59	1995	22	1.0(22)	_	_	_	_	_	1.0(3)	_	_	_	_	*#2 (1)*	Pasteur et al. 2001
		Saigon	60	1995	23	1.0(23)	_	_	_	_	_	1.0(3)	_	_	_	_	*#2 (1)*	Pasteur et al. 2001
	Thailand																	
		Bangkok	61	2012	20	1.0(20)	_	_	_	_	_	1.0(3)	_	_	_	_	*#2 (1)*	This study
		Thaï	62	1992	24	1.0(24)	_	_	_	_	_	1.0(3)	_	_	_	_	*#2 (1)*	This study
	Indonesia																	
		KUP	63	2003	9	1.0(9)	_	_	_	_	_	1.0(3)	_	_	_	_	*#2 (1)*	Fonseca et al. 2006
	Kazakhstan																	
		Alma-Ata	104	2010	15	_	_	_	1.0(15)	_	_	ND	ND	ND	ND	ND	*ND*	This study
	Uzbekistan																	
		UZB	101	2004	6	_	_	_	1.0(6)	_	_	_	_	1.0(6)	_	_	*#9 (1)*	Turell et al. 2004
Oceania																		
	Australia																	
		Aus (LL)	64	2004	1	_	1.0(1)	_	_	_	_	ND	ND	ND	ND	ND	*#5 (1)*	Duron et al. 2005

		Killcare (LL)	65	1993	1	_	_	_	_	1.0(1)	_	_	_	1.0(1)	_	_	*#2 1)*	Guillemaud et al. 1997
	New Caledonia																	
		Poindimié	66	2012	13	1.0(13)	_	_	_	_	_	0.5(2)	_	_	_	0.5(2)	*#3 (1)*	This study
	French Polynesia																	
		Moorea	67	1992	14	1.0(14)	_	_	_	_	_	1.0(3)	_	_	_	_	*#3 (1)*	Pasteur et al. 2005
		Tabu	68	1992	3	1.0(3)	_	_	_	_	_	1.0(1)	_	_	_	_	*#3 (1)*	Pasteur et al. 2005
	Hawai																	
		Lihue	90	2001	11	_	_	_	_	1.0(11)	_	1.0(11)	_	_	_	_	*#2 (1)*	Fonseca et al. 2006
America																		
	Brazil																	
		Recife	69	1995	16	1.0(16)	_	_	_	_	_	1.0(2)	_	_	_	_	*#2 (1)*	Duron et al. 2005
		Brasilia	70	1993	16	1.0(16)	_	_	_	_	_	1.0(2)	_	_	_	_	*#2 (1)*	Guillemaud et al. 1997
	Guyana																	
		Matoury	71	2011	23	1.0(23)	_	_	_	_	_	1.0(3)	_	_	_	_	*#2 (1)*	This study
	Venezuela																	
		Cimetière Printemps	72	1997	22	_	_	1.0(22)	_	_	_	1.0(3)	_	_	_	_	*#6 (1)*	This study
	Costa Rica																	
		Guacimo	73	2006	23	_	_	1.0(23)	_	_	_	1.0(3)	_	_	_	_	*#6 (2)*	This study
		Guapiles	74	2006	20	0.1(1)	_	0.9(19)	_	_	_	1.0(3)	_	_	_	_	*#2 (1), #6 (1)*	This study
		Puerto Viejo de Talamanque	75	2006	23	0.1(1)	_	0.9(22)	_	_	_	1.0(3)	_	_	_	_	*#3 (1), #6 (1)*	This study
	Galapagos																	
		Gal	76	2004	10	_	_	1.0(10)	_	_	_	1.0(10)	_	_	_	_	*ND*	Fonseca et al. 2006
	Martinique																	
		Martinique (LL)	77	2003	1	1.0(1)	_	_	_	_	_	1.0(1)	_	_	_	_	*#3 (1)*	Guillemaud et al. 1997
	Jamaica																	
		JM	78	2004	11	0.7(8)	_	0.3(3)	_	_	_	1.0(11)	_	_	_	_	*#2 (1)*	Fonseca et al. 2006
	Puerto Rico																	
		Rico	79	1992	22	_	_	1.0(22)	_	_	_	1.0(3)	_	_	_	_	*#6 (1)*	This study
	Haiti																	
		Gonaives	80	1991	24	_	_	1.0(24)	_	_	_	1.0(3)	_	_	_	_	*#6 (1)*	This study
	Mexico																	
		Chiapas	81	1998	2	_	_	1.0(2)	_	_	_	1.0(2)	_	_	_	_	*ND*	Fonseca et al. 2006

		Jalisco (LL)	82	1998	1	_	_	1.0(1)	_	_	_	1.0(1)	_	_	_	_	*ND*	Fonseca et al. 2006
	USA																	
		Miami (LL)	83	1991	1	_	_	1.0(1)	_	_	_	1.0(1)	_	_	_	_	*#6 (1)*	Duron et al. 2005
		New Orleans	84	1999	11	0.3(3)	_	0.7(8)	_	_		1.0(12)					*ND*	Fonseca et al. 2004
		Madeira	85	2010	14	_	_	1.0(14)	_	_	_	_	1.0(1)	_	_	_	*#6 (1)*	This study
		MaClo (LL)	86	1984	1	_	_	1.0(1)	_	_	_	1.0(1)	_	_	_	_	*#6 (1)*	Duron et al. 2006
		Slab (LL)	87	1950	1	_	_	1.0(1)	_	_	_	1.0(1)	_	_	_	_	*#7 (1)*	Georghiou et al. 1966
		TEM-R (LL)	88	1978	1	_	_	1.0(1)	_	_	_	1.0(1)	_	_	_	_	*#6 (1)*	Georghiou & Pasteur 1978
		EDIT (LL)	89	1988	1	_	_	1.0(1)	_	_	_	ND	ND	ND	ND	ND	*ND*	Guillemaud et al. 1999
		SELAX-B (LL)	92	1984	1	_	_	1.0(1)	_	_	_	ND	ND	ND	ND	ND	*ND*	Duron et al. 2005
		TRANS-P (LL)	93	1975	1	_	_	1.0(1)	_	_	_	ND	ND	ND	ND	ND	*ND*	Priester & Georghiou 1978
		PRO-R (LL)	94	1963	1	_	_	1.0(1)	_	_	_	ND	ND	ND	ND	ND	*ND*	Georghiou et al. 1966
		New York FT	96	2002	24	_	_	1.0(24)	_	_	_	_	0.5(1)	0.5(1)	_	_	*#6 (1)*	This study
		New York Sewer	97	2002	21	_	_	1.0(21)	_	_	_	_	_	1.0(3)	_	_	*#6 (1)*	This study
		Albany	98	2001	7	_	_	1.0(7)	_	_	_	_	1.0(7)	_	_	_	*#6 (1)*	Fonseca et al. 2004
		Minnessota (LL)	99	1987	1	_	_	1.0(1)	_	_	_	_	1.0(1)	_	_	_	*#6 (1)*	Duron et al. 2005
Europe																		
	Belgium																	
		Slim-mol	134	2012	26	_	_	1.0(26)	_	_	_	_	_	1.0(6)	_	_	*ND*	This study
		Bruges-B (LL)	135	1991	1	_	_	_	1.0(1)	_	_	_	_	1.0(1)	_	_	*#9 (1)*	Raymond et al. 1996
	Netherlands																	
		Heteren (LL)	133	1992	1	_	_	_	1.0(1)	_	_	_	_	1.0(1)	_	_	*#9 (1)*	Weill et al. 2003
	Spain																	
		Palmier	156	1996	23	_	0.3(6)	0.7(17)	_	_	_	_	1.0(1)	_	_	_	*#5 (2), #6 (1)*	Eritja & Chevillon 1999
		El Palmar	162	2005	19	1.0(19)	_	_	_	_	_	_	0.5(1)	0.5(1)	_	_	*#2 (1)*	Duron et al. 2007
		El Palmar-A (LL)	163	2005	1	1.0(1)	_	_	_	_	_	ND	ND	ND	ND	ND	*#2 (1)*	Duron et al. 2007
		El Palmar-B (LL)	164	2005	1	1.0(1)	_	_	_	_	_	ND	ND	ND	ND	ND	*#2 (1)*	Duron et al. 2007
		Castilnuevo (LL)	161	2011	1	_	_	1.0(1)	_	_	_	_	1.0(1)	_	_	_	*#6 (1)*	This study
		Lotto	157	1996	25	_	0,3(8)	0,7(17)	_	_		_	_	1.0(3)	_	_	*#5 (3), #6 (1), #9 (1)*	Eritja & Chevillon 1999
		Menthe	159	1996	27	_	0,48(13)	0,48(13)	0,04(1)	_	_	_	1.0(3)	_	_	_	*#5 (1), #6 (2), #9 (1)*	Eritja & Chevillon 1999
		Viladecans (LL)	158	2011	1	_	_	1.0(1)	_	_	_	_	_	1.0(1)	_	_	*#6 (1)*	This study
		Molins de Rei	160	2011	8	_	0.1(1)	0.8(6)	0.1(1)	_	_	_	0.7(2)	0.3(1)	_	_	*#5 (1), #6 (1), #9 (1)*	This study

Germany																	
	Berlin Park	132	2009	8	_	1.0(8)	_	_	_	_	ND	ND	ND	ND	ND	*ND*	This study
	GeA	130	2003	11	_	1.0(11)	_	_	_	_	_	1.0(11)	_	_	_	*#5 (1)*	Fonseca et al. 2004
	GeB	131	2003	11	_	0.3(3)	0.6(7)	0.1(1)	_	_	_	_	1.0(11)	_	_	*#5 (1), #6 (1), #9(1)*	Fonseca et al. 2004
France																	
	Bifa-A (LL)	146	2002	1	1.0(1)	_	_	_	_	_	_	1.0(1)	_	_	_	*#3 (1)*	Duron et al. 2006
	Bifa-B (LL)	148	2002	1	_	_	1.0(1)	_	_	_	_	1.0(1)	_	_	_	*#6 (1)*	Duron et al. 2006
	Lavar (LL)	143	2003	1	_	1.0(1)	_	_	_	_	_	1.0(1)	_	_	_	*#5 (1)*	Duron et al. 2005
	PopD	140	1991	22	_	0.8(18)	0.2(4)	_	_	_	_	1.0(4)	_	_	_	*#5 (2), #6 (2)*	This study
	Canejan	154	1990	24	_	0.8(20)	0.2(4)	_	_	_	_	1.0(4)	_	_	_	*#5 (1), #6 (1)*	This study
	BOB	145	1993	16	_	0.7(12)	0.3(4)	_	_	_	_	1.0(1)	_	_	_	*#5 (1), #7 (1)*	This study
	Triolet	151	2011	24	_	_	1.0(24)	_	_	_	_	_	1.0(3)	_	_	*#11 (1)*	This study
	Triolet-2 (LL)	149	2011	1	_	_	1.0(1)	_	_	_	ND	ND	ND	ND	ND	*ND*	This study
	Triolet-7 (LL)	150	2011	1	_	_	1.0(1)	_	_	_	ND	ND	ND	ND	ND	*ND*	This study
	Dijon	139	1989	20	_	1.0(20)	_	_	_	_	_	1.0(2)	_	_	_	*#5 (1)*	This study
	Ganges	147	2009	24	_	0.7(16)	0.3(8)	_	_	_	ND	ND	ND	ND	ND	*#5 (1), #6 (3)*	This study
	Catelmaurou	153	2011	12	_	1.0(12)	_	_	_	_	_	1.0(3)	_	_	_	*#5 (1)*	This study
	Vilenne	138	2003	9	_	1.0(9)	_	_	_	_	_	1.0(3)	_	_	_	*#5 (1)*	This study
	Maurin	144	2001	24	_	0.6(15)	0.34(9)	_	_	_	_	0.7(3)	0.3(1)	_	_	*#5 (1), #6 (1)*	This study
	Merville	137	2011	2	_	1.0(2)	_	_	_	_	_	1.0(1)	_	_	_	*#5 (1)*	This study
	Arras	136	2011	7	_	_	1.0(7)	_	_	_	_	_	1.0(3)	_	_	*#6 (1)*	This study
	Vercors	142	1991	4	_	0.7(3)	0,3(1)	_	_	_	ND	ND	ND	ND	ND	*#5 (1), #6 (1)*	This study
	Carbasse	152	1996	25	_	0,6(16)	0,4(9)	_	_	_	_	1.0(3)	_	_	_	*#5 (2), #6(1)*	This study
	Oléron	155	1991	16	_	0.9(15)	0.1 (1)	_	_	_	_	0.7(2)	0.3(1)	_	_	*#5 (2), #6 (1)*	This study
Portugal																	
	Praias	168	1993	7	1.0(7)	_	_	_	_	_	_	0.5(1)	0.5(1)	_	_	*#2 (1)*	Bourguet et al. 1996
	Ferreira	166	1993	6	_	0.8(5)	0.2(1)	_	_	_	_	1.0(3)	_	_	_	*#5 (1), #6 (1)*	Duron et al. 2005
	Mitra	165	1993	5	0.2(1)	0.4(2)	0.4(2)	_	_	_	_	1.0(3)	_	_	_	*#2 (1), #5 (1), #6 (1)*	Duron et al. 2005
	Bodes	183	1994	3	_	_	_	1.0(3)	_	_	_	_	1.0(2)	_	_	*#9 (1)*	Duron et al. 2005
	Madera	184	unknown	2	_	_	_	1.0(2)	_	_	_	0.5(1)	0.5(1)	_	_	*#9 (1)*	This study
	Seia	167	2011	23	0.2(4)	0.2(4)	0.5(12)	_	_	0.1(3)	_	1.0(5)	_	_	_	*#5 (3), #6 (1)*	This study
United Kingdom																	

	Menstrie	177	2001	7	1.0(7)	_	_	_	_	_	_	_	1.0(2)	_	_	*#3 (1)*	Fonseca et al. 2004
	Wedmore	181	2001	10	_	1.0(10)	_	_	_	_	_	1.0(2)	_	_	_	*ND*	Fonseca et al. 2006
	Liverpool	180	2007	2	_	1.0(2)	_	_	_	_	_	1.0(1)	_	_	_	*#5 (1)*	This study
	Rothamsted	182	1991	5	_	0.6(3)	0.4(2)	_	_	_	_	1.0(1)	_	_	_	*#5 (1)*	Duron et al. 2005
	Quest	178	2002	6	_	1.0(6)	_	_	_	_	_	1.0(2)	_	_	_	*#5 (1)*	Fonseca et al. 2004
	Willow	179	2001	8	_	0.9(7)	0.1(1)	_	_	_	_	1.0(2)	_	_	_	*#5 (1)*	Duron et al. 2005
Italia																	
	Cremone	129	2000	19	0,1(1)	_	0.1(2)	0.8(16)	_	_	_	1.0(3)	_	_	_	*#6 (2), #8 (3)*	This study
	Soragna	128	2011	23	_	_	_	1.0(23)	_	_	_	1.0(3)	_	_	_	*#9 (1)*	This study
	St Pietro	127	unknown	22	_	_	_	1.0(22)	_	_	_	1.0(3)	_	_	_	*#9 (1)*	This study
	Padova	126	unknown	8	_	_	_	1.0(8)	_	_	_	1.0(2)	_	_	_	*#8 (1)*	Bourguet et al. 1997
	CAA (LL)	125	unknown	1	_	_	_	1.0(1)	_	_	_	_	1.0(1)	_	_	*#9 (1)*	This study
	Latina	123	2011	24	1.0(24)	_	_	_	_	_	_	1.0(3)	_	_	_	*#3 (1)*	This study
	Roma	124	2011	6	1.0(6)	_	_	_	_	_	_	_	1.0(3)	_	_	*#3 (1)*	This study
Greece																	
	Thessaloniki	122	2010	11	_	1.0(11)	_	_	_	_	_	1.0(3)	_	_	_	*#5 (1)*	This study
	Plage	119	2002	8	1.0(8)	_	_	_	_	_	_	1.0(2)	_	_	_	*#3 (1)*	Duron et al. 2005
	Lamia	121	2011	20	_	0.2(4)	_	0.8(16)	_	_	_	1.0(2)	_	_	_	*#5 (1) , #9(1)*	This study
	Kol (LL)	120	2002	1	1.0(1)	_	_	_	_	_	ND	ND	ND	ND	ND	*#3 (1)*	Duron et al. 2005
Cyprus																	
	Keo-A (LL)	115	2003	1	_	1.0(1)	_	_	_	_	_	1.0(1)	_	_	_	*#5 (1)*	Duron et al. 2006
	Keo-B (LL)	116	2003	1	_	1.0(1)	_	_	_	_	_	1.0(1)	_	_	_	*#5 (1)*	Duron et al. 2006
	Néné	117	2004	24	_	1.0(24)	_	_	_	_	_	0.3(1)	0.7(2)	_	_	*#5 (1)*	Duron et al. 2005
Switzerland																	
	Perrin	141	2004	20	_	1.0(20)	_	_	_	_	_	1.0(3)	_	_	_	*#5 (1)*	Duron et al. 2005
Russia																	
	Moscou	107	2006	10	_	1.0(10)	_	_	_	_	_	1.0(2)	_	_	_	*#5 (1)*	This study
	Krasnodar	106	2006	10	_	0.6(6)	_	_	0.4(4)	_	_	1.0(3)	_	_	_	*#2 (1), #5 (1)*	This study
	Ekaterinburg	102	2005	6	–	–	–	1.0(6)	–	–	ND	ND	ND	ND	ND	*ND*	This study

	Tomsk	103	2003	9	–	–	–	1.0(9)	_	_	ND	ND	ND	ND	ND	*ND*	This study
	Volgograd	100	2006	3	_	_	_	0.3(1)	0.67(2)	_	ND	ND	ND	ND	ND	*ND*	This study
TOTAL				1935	0.43(823)	0.17(326)	0.21(414)	0.13(261)	0.057(108)	0.003(3)	0.49(201)	0.29(118)	0.16(64)	0.05(20)	0.01(6)	Total: 184	

Table S1. Detailed results of the screen of *Culex pipiens* populations. LL, laboratory lines; ND, not determined.

References

Atyame CM, Pasteur N, Dumas E, Tortosa P, Tantely ML, Pocquet N, Licciardi S, Bheecarry A, Zumbo B, Weill M, Duron O : **Cytoplasmic incompatibility as a means of controlling Culex pipiens quinquefasciatus mosquito in the islands of the south-western Indian Ocean**. PLoS Negl Trop Dis. 2011, Dec;5(12):e1440.

Beyssat-Arnaouty V, Mouchès C, Georghiou GP, Pasteur N : **Detection of organophosphate detoxifying esterases by dot-blot immunoassay in *Culex* mosquitoes.** ***Journal of American MosquitoControl Association***, 1989, 5, 196–200.

Erija R, Chevillon C: **Interruption of chemical mosquito control and evolution of insecticide resistance genes in *Culex pipiens* (Diptera: Culicidae)**. *Journal of Medical Entomology*, 1999, **36**, 41–49.

Fonseca DM, Keyghobadi N, Malcolm CA, Mehmet C, Schaffner F, Mogi M, Fleischer RC, Wilkerson RC: **Emerging vectors in the Culex pipiens complex**. *Science* 2004, **303**:1535–1538.

Fonseca DM, Smith JL, Wilkerson RC, Fleischer RC: **Pathways of expansion and multiple introductions illustrated by large genetic differentiation among worldwide populations of the southern house mosquito**. *Am J Trop Med Hyg* 2006, **74**:284–289.

Fonseca D, Smith J, Kim H, Mogi M: **Population genetics of the mosquito *Culex pipiens pallens* reveals sex-linked asymmetric introgression by *Culex quinquefasciatus***. *Infect Genet Evol*, **2009**, 1197–1203.

Georghiou GP, Metcalf RL, Gidden FE: **Carbamate resistance in mosquitoes: selection of *Culex pipiens fatigans* Wied (*Culex quinquefasciatus*) for resistance to Baygon**. *Bulletin of the World Health Organization*, 1966, 35, 691–708.

Guillemaud T, Raymond M, Tsagkarakou A, Bernard C, Rochard P, Pasteur N: **Quantitative variations and selection of esterase gene amplification in *Culex pipiens***. *Heredity*, 1999, 83, 87–99.

Klasson L, Walker T, Sebaihia M, Sanders MJ, Quail MA, Lord A, Sanders S, Earl J, O'Neill SL, Thomson N, Sinkins SP, Parkhill J : **Genome evolution of Wolbachia strain wPip from the Culex pipiens group**. Mol Biol Evol. 2008, Sep;25(9):1877-87.

Magnin M, Marboutin E, Pasteur N : **Insecticide resistance in *Culex quinquefasciatus* (Diptera: Culicidae) in West Africa**. *Journal of Medical Entomology*, 1988, 25, 99–104.

Pasteur N, Marquine M, Hoang TH, Sinh-Nam V, Failloux A-B: **Overproduced esterases in *Culex pipiens quinquefasciatus* (Diptera:Culicidae) from Vietnam**. *Journal of Medical Entomology*, 2001, 38, 740–745.

Priester TM, Georghiou GP : **Induction of high resistance to permethrin in *Culex pipiens quinquefasciatus***. *Journal of Economical Entomology*, 1978, 71, 197–200.

Qiao C-L, Raymond M: **The same esterase B1 haplotype is amplified in insecticide resistant mosquitoes of the *Culex pipiens* complex from the Americas and China.** *Heredity*, 1995, 74, 339–345.

Raymond M, Qiao C-L, Callaghan A: **Esterase polymorphism in insecticide susceptible populations of the mosquito *Culex pipiens***. *Genetical Research*, 1996, 67, 19–26.

Salzberg SL, Puiu D, Sommer DD, Nene V, Lee NH : **Genome sequence of the Wolbachia endosymbiont of Culex quinquefasciatus JHB**. J Bacteriol. 2009, Mar;191(5):1725.

Sinkins, S. P., T. Walker, A. R. Lynd, A. R. Steven, B. L. Makepeace, H. C. J.Godfray, and J. Parkhill: ***Wolbachia* variability and host effects on crossing type in *Culex* mosquitoes**. Nature, 2005, 436:257–260.

Turell MJ, Mores CN, Dohm DJ, Komilov N, Paragas J, Lee JS, Shermuhemedova D, Endy TP, Kodirov A, Khodjaev S: **Laboratory transmission of Japanese encephalitis and West Nile viruses by molestus form of Culex pipiens (Diptera: Culicidae) collected in Uzbekistan in 2004**. J Med Entomol. 2006, Mar;43(2):296-300.

Weill M, Lutfalla G, Mogensen K, Chandre F, Berthomieu A, Berticat C, Pasteur N, Philips A, Fort P, Raymond M *:* **Insecticide resistance in mosquito vectors**. *Nature*, 2003, 423, 136–137.

Gene	Locus tag in *w*Pip(Pel) genome	Putative product	Primer (5'-3')	Size (pb)	Anneling temperature (°C)	Reference
Wolbachia						
ank2	Wpa_0652	Ankyrin domain protein	F-CTTCTTCTGTGAGTGTACGT<br>R2-TCCATATCGATCTACTGCGT	313-511	52	(Duron et al. 2007)
pk1	Wpa_0256 (1)<br>Wpa_0313 (2)<br>Wpa_1306 (3)	Ankyrin domain protein	F-CCACTACATTGCGCTATAGA<br>R-ACAGTAGAACTACACTCCTCCA	1,334-1,349	52	(Sinkins et al. 2005)<br>(Duron et al. 2007)
MutL	Wpa_0278	DNA mismatch repair protein	F- ACTTCATTGCCCTTCCAGCT<br>R -GGCATCAAATTAAGGGACA	1000-1,063	58	(Atyame et al. 2011)
GP15	Wpa_1322	Phage related probable secretory protein	F1-ACCATTACAGAACTTGAGGA<br>R1-TAGACGTTCATAGGCAACCA	1,511-1,538	52	(Duron, Fort, and Weill 2006)
RepA	Wpa_1312	Phage related replication protein	F1-ACTTTAGAGGGGTGCTTTCT<br>R2- ACAAACAACGGCACAGATT	583-1,501	52	(Duron et al. 2005)
Culex pipiens						
mitochondrial						
cytb		Cytochrome b	F-CTTTATAGTAACTGTAAAAATTAC<br>R-ACTAAAGGATTAGCGGAATG	852	52	(Atyame et al. 2011)
ND2		NADH dehydrogenase subunit 2	F-TGGCTTGGTGCTTGAATAGGG<br>R-AATGGCTGAAGTTTAGGCGAT	1,160	56	(Atyame et al. 2011)
ND5		NADH dehydrogenase subunit 5	F6981-GAATAAAACCCTGCTAAAAAAG<br>R8112-GATTTGTGGTGTCAATGATA	1,132	52	(Atyame et al. 2011)
nuclear						
ace2		Acethylcholinesterase 2	F1457-GAGGAGATGTGGAATCCCAA<br>R1246-TGGAGCCTCCTCTTCACGGC	700	50	(Bourguet et al. 1998)
CQ11		CQ11 locus	CQ11F2-GATCCTAGCAAGCGAGAAC<br>molCQ11R-CCCTCCAGTAAGGTATCAAC<br>pipCQ11R-CATGTTGAGCTTCGGTGAA	CQ11F2-molCQ11R: 250<br>CQ11F2-pipCQ11R: 200	54	(Bahnck and Fonseca 2006)

Table S2. List of primers and gene features.

References

Atyame CM, Delsuc F, Pasteur N, Weill M, Duron O: **Diversification of Wolbachia endosymbiont in the Culex pipiens mosquito.** *Molecular biology and evolution* , **28**:2761–72.

Bahnck CM, Fonseca DM: **Rapid assay to identify the two genetic forms of Culex (Culex) pipiens L. (Diptera: Culicidae) and hybrid populations.** *The American journal of tropical medicine and hygiene* 2006, **75**:251–5.

Bourguet D, Fonseca D, Vourch G, Dubois MP, Chandre F, Severini C, Raymond M: **The acetylcholinesterase gene ace: a diagnostic marker of the pipiens and quinquefasciatus forms of the Culex pipiens complex**. *J Amer Mosq Control Assoc* 1998, **14**:390–396.

Duron O, Lagnel J, Raymond M, Bourtzis K, Fort P, Weill M: **Transposable element polymorphism of Wolbachia in the mosquito Culex pipiens: evidence of genetic diversity, superinfection and recombination**. *Mol Ecol* 2005, **14**:1561–1573.

Duron O, Fort P and Weill M: **Hypervariable prophage WO sequences describe an unexpected high number of *Wolbachia* variants in the mosquito *Culex pipiens***. Proceedings of the Royal Society of London - Series B, 2006, 273: 495-502.

Duron O, Boureux A, Echaubard P, Berthomieu A, Berticat C, Fort P, Weill M: **Variability and expression of ankyrin domain genes in Wolbachia variants infecting the mosquito Culex pipiens**. *J Bacteriol* 2007, **189**:4442–4448.

Duron O, Bouchon D, Boutin S, Bellamy L, Zhou L, Engelstadter J, Hurst GD: **The diversity of reproductive parasites among arthropods: Wolbachia do not walk alone**. *BMC Biology* 2008, **6**:27.

Sinkins, S. P., T. Walker, A. R. Lynd, A. R. Steven, B. L. Makepeace, H. C. J.Godfray, and J. Parkhill: ***Wolbachia* variability and host effects on crossing type in *Culex* mosquitoes**. Nature, 2005, 436:257–260.

mtDNA haplotypes	Gene, position																						
	cytb										*ND2*				*ND5*								
	10,502	10,554	10,715	10,758	10,887	10,918	10,919	10,943	10,952	11,118	256	470	591	660	7,061	7,106	7,280	7,341	7,345	7,571	7,824	7,826	7,927
haplotype#1	A	A	C	G	G	G	G	C	A	A	A	T	T	T	T	A	T	G	C	G	A	C	A
haplotype#2	-	G	-	-	-	-	-	-	-	-	G	-	-	-	-	-	-	-	-	-	G	-	-
haplotype#3	-	G	-	-	A	-	-	-	-	-	G	-	C	-	A	-	-	-	-	-	G	-	-
haplotype#4	-	G	T	-	A	-	-	-	-	-	G	-	C	-	-	-	-	-	-	-	G	-	-
haplotype#5	-	G	-	-	-	-	-	-	-	G	G	-	-	A	-	G	-	-	-	-	G	T	-
haplotype#6	G	G	-	-	-	-	-	-	G	G	G	-	-	A	-	-	-	-	-	-	G	T	-
haplotype#7	G	G	-	-	-	-	-	-	-	G	G	-	-	A	-	-	-	-	-	-	G	T	-
haplotype#8	-	G	-	-	-	A	-	-	-	-	G	-	-	A	-	-	-	A	-	A	G	T	-
haplotype#9	-	G	-	-	-	-	A	-	-	-	G	-	-	A	-	-	-	-	-	-	G	T	-
haplotype#10	-	G	-	A	-	-	-	T	-	-	G	-	-	A	-	-	-	-	-	-	G	T	-
haplotype#11	-	G	-	-	-	-	-	T	-	-	G	-	-	A	-	-	-	-	-	-	G	T	-

Table S3. Nucleotide polymorphism in the *cytb*, *ND2* and *ND5* mitochondrial genes. Only polymorphic sites are represented and a dash indicates similarity with the top sequence. Position expressed in nucleotides bases on the complete mitochondrial sequence of Pel *Culex pipiens* line (Klasson *et al.* 2008).

Reference

Klasson L, Walker T, Sebaihia M, Sanders MJ, Quail MA, Lord A, Sanders S, Earl J, O'Neill SL, Thomson N, Sinkins SP, Parkhill J: **Genome evolution of *Wolbachia* strain wPip from the *Culex pipiens* group**. Mol Biol Evol. 2008, Sep;25(9):1877-87.

	Lihue, 90	JM, 78	KNY, 12	KUP, 63	NOR, 84	GAL, 76	CPJ, 52	ABA, 53	GeB, 131	JRD, 114	GeA, 130
JM, 78	0.114										
KNY, 12	0.077	0.106 *									
KUP, 63	0.120	0.142 *	0.056 *								
NOR, 84	0.095	0.068	0.132 *	0.160 *							
GAL, 76	0.144	0.251 *	0.131 *	0.080	0.213 *						
CPJ, 52	0.326 *	0.236 *	0.241 *	0.298 *	0.271 *	0.427 *					
ABA, 53	0.318 *	0.257 *	0.186 *	0.264 *	0.306 *	0.402 *	0.027				
GeB, 131	0.610 *	0.501 *	0.501 *	0.529 *	0.541 *	0.660 *	0.471 *	0.471 *			
JRD, 114	0.463 *	0.372 *	0.359 *	0.390 *	0.423 *	0.552 *	0.346 *	0.331 *	0.154 *		
GeA, 130	0.272 *	0.232 *	0.207 *	0.238 *	0.290 *	0.390 *	0.187 *	0.165 *	0.340 *	0.230 *	
Wed, 181	0.330 *	0.255 *	0.247 *	0.276 *	0.321 *	0.439 *	0.204 *	0.185 *	0.377 *	0.223 *	0.012

Table S4. Pair-wise FST values among mosquito populations (*quinquefasciatus*: populations 78, 12, 63, 84, 76 and 90 in Table S1, *pallens*: 52 and 53, *molestus*: 131 and 114, *pipiens*: 130 and 181). *, significant F_{ST} values after Bonferroni correction.

Figure legends

Figure S1. Identification of *ank2* and *pk1* allelic profiles. (A) *Hinf*I digestion of the *ank2* PCR products allowed discrimination of five alleles (a to e): a (one RFLP fragment: 313bp), b (217, 195, 98bp), c (293, 217bp), d (217, 195bp) and e (415bp). (B) *Taq*I digestion of the *pk1* PCR products allowed discrimination of four specific *w*Pip alleles (alleles a and e have the same fragment sizes): a/e (903, 430bp), b (669, 665bp), c (851, 498bp) and d (497, 251, 107bp). (C) *PstI* digestion of the *pk1* PCR products allowed discrimination alleles a (903, 303, 141bp) and e (903, 430bp).

Figure S2. *mtDNA* phylogeny constructed using Maximum likelihood method based on concatenated sequences of *cytb, ND2* and *ND5* genes. mtDNA haplotypes originally described by Atyame *et al.* (2011b) are marked by full circles. Triangles show the seven specimens presenting incongruences between *w*Pip infection and mtDNA haplotypes. Numbers on branches indicate percentage bootstrap support for major branches (500 replicates). The scale bar indicates the number of substitutions.

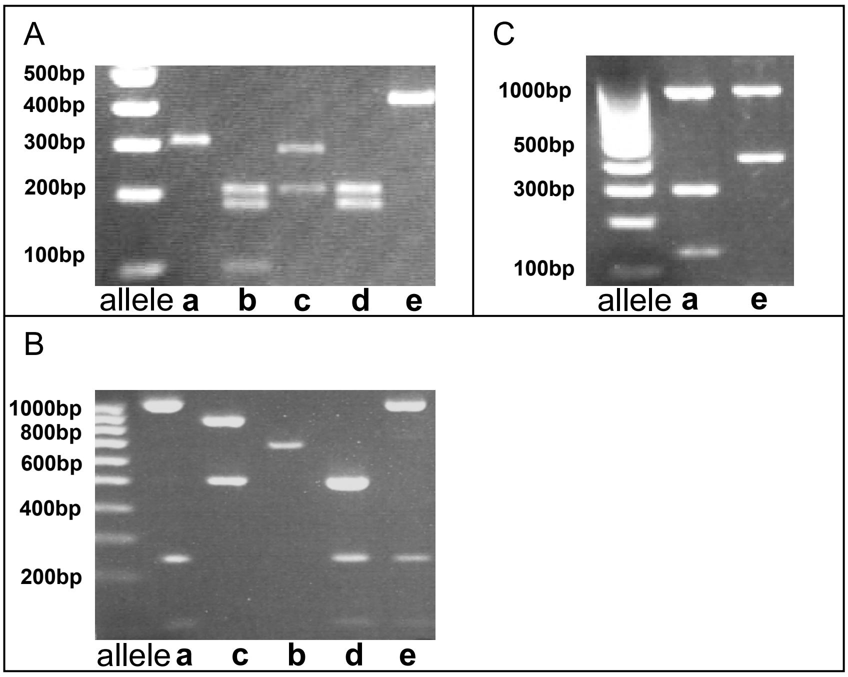
A
500bp
400bp
300bp
200bp
100bp
allele a b c d e
C
1000bp
500bp
300bp
100bp
allele a e
B
1000bp
800bp
600bp
400bp
200bp
allele a c b d e

Figure S1

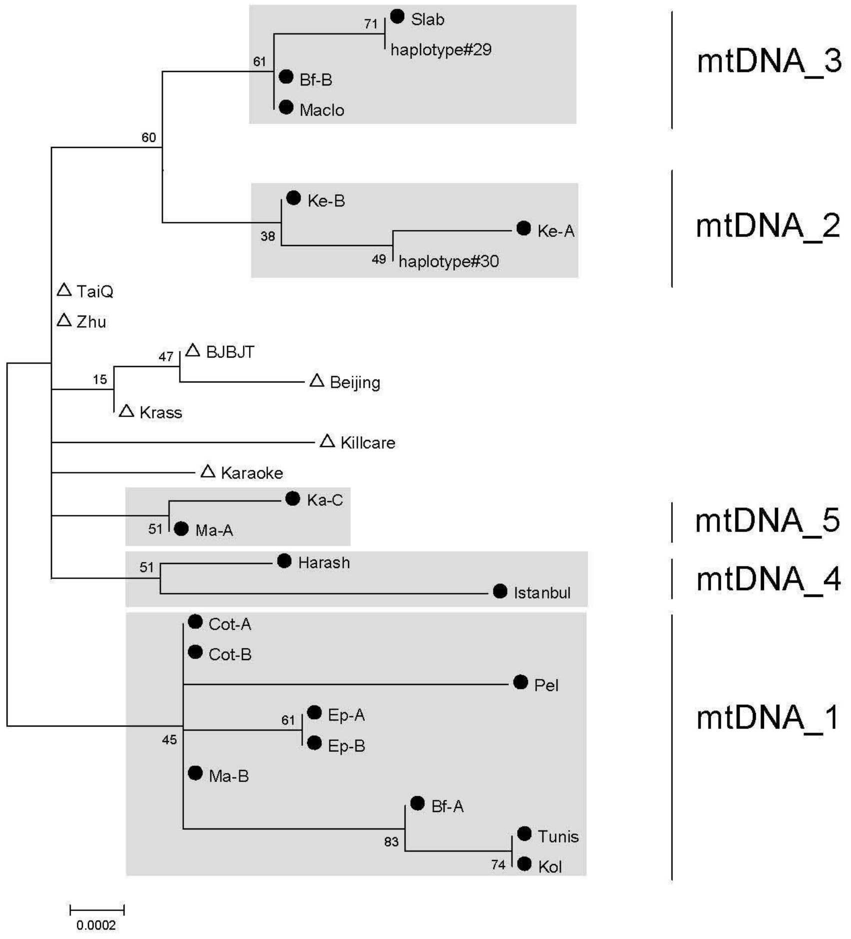
71
Slab
haplotype#29
61
Bf-B
Maclo
60
mtDNA_3
Ke-B
Ke-A
38
49
haplotype#30
mtDNA_2
TaiQ
Zhu
47
BJBJT
15
Beijing
Krass
Killcare
Karaoke
Ka-C
51
Ma-A
mtDNA_5
51
Harash
Istanbul
mtDNA_4
Cot-A
Cot-B
Pel
61
Ep-A
45
Ep-B
Ma-B
Bf-A
83
Tunis
74
Kol
mtDNA_1
0.0002

Figure S2
